# 5,5′,5′′-Triphenyl-2,2′,2′′-[2,4,6-tri­methyl­benzene-1,3,5-triyltris(methyl­idene­sulfanedi­yl)]tris­(1,3,4-oxadiazole)

**DOI:** 10.1107/S1600536810040730

**Published:** 2010-10-20

**Authors:** Wei Wang, Yan Gao, Ming Ji, Hong-guo Yao, Hong Qiu

**Affiliations:** a School of Perfume and Aroma Technology, Shanghai Istitute of Technology, Shanghai 200235, People’s Republic of China; b School of Chemical Engineering, University of Science and Technology LiaoNing, Anshan 114051, People’s Republic of China; c Liaoyang Supervision and Examination Station of Product Quality, Liaoning Liaoyang 111000, People’s Republic of China

## Abstract

In the title compound, C_36_H_30_N_6_O_3_S_3_, the phenyl rings are twisted from the attached oxadiazole rings in the three arms by 1.5(2), 2.4 (2) and 25.7 (2)°. The crystal packing exhibits weak inter­molecular C—H⋯N inter­actions.

## Related literature

For general background to 1,3,4-oxadiazole derivatives, see Al-Talib *et al.* (1990 ▶); Wang *et al.* (2005 ▶) and to thio-based ligands with a multi-armed tripodal geometry, see: PrakashaReddy & Pedireddi (2007 ▶). For the crystal structure of an Ag complex with a related oxadiazole derivative, see: Zhang *et al.* (2007 ▶).
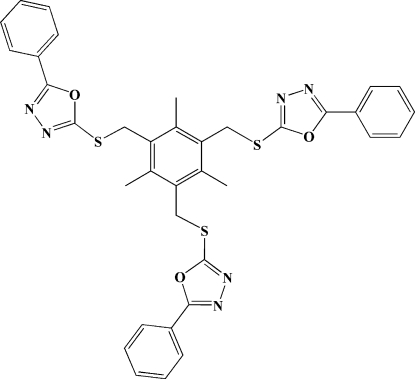


## Experimental

### 

#### Crystal data

C_36_H_30_N_6_O_3_S_3_
*M* *_r_* = 690.84
Monoclinic, 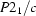

*a* = 19.870 (4) Å
*b* = 9.1305 (18) Å
*c* = 18.557 (4) Å
β = 107.00 (3)°
*V* = 3219.6 (11) Å^3^
*Z* = 4
Mo *K*α radiation
μ = 0.28 mm^−1^
*T* = 113 K
0.22 × 0.20 × 0.10 mm

#### Data collection

Rigaku Saturn CCD area-detector diffractometer
Absorption correction: multi-scan (*CrystalClear*; Rigaku/MSC, 1999 ▶) *T*_min_ = 0.941, *T*_max_ = 0.973
23119 measured reflections
5664 independent reflections
4490 reflections with *I* > 2σ(*I*)
*R*_int_ = 0.051

#### Refinement

*R*[*F* ^2^ > 2σ(*F* ^2^)] = 0.049
*wR*(*F* ^2^) = 0.149
*S* = 1.10
5664 reflections
437 parameters
H-atom parameters constrained
Δρ_max_ = 0.35 e Å^−3^
Δρ_min_ = −0.37 e Å^−3^

### 

Data collection: *CrystalClear* (Rigaku/MSC, 1999 ▶); cell refinement: *CrystalClear*; data reduction: *CrystalClear*; program(s) used to solve structure: *SHELXS97* (Sheldrick, 2008 ▶); program(s) used to refine structure: *SHELXL97* (Sheldrick, 2008 ▶); molecular graphics: *SHELXTL* (Sheldrick, 2008 ▶); software used to prepare material for publication: *SHELXTL*.

## Supplementary Material

Crystal structure: contains datablocks global, I. DOI: 10.1107/S1600536810040730/cv2772sup1.cif

Structure factors: contains datablocks I. DOI: 10.1107/S1600536810040730/cv2772Isup2.hkl

Additional supplementary materials:  crystallographic information; 3D view; checkCIF report

## Figures and Tables

**Table 1:** Hydrogen-bond geometry (Å, °)

*D*—H⋯*A*	*D*—H	H⋯*A*	*D*⋯*A*	*D*—H⋯*A*
C31—H31⋯N5^i^	0.95	2.55	3.338 (3)	141
C24—H24⋯N2^ii^	0.95	2.57	3.394 (4)	146

Symmetry codes: (i) 
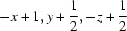
; (ii) 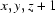.

**Fig. 1.**
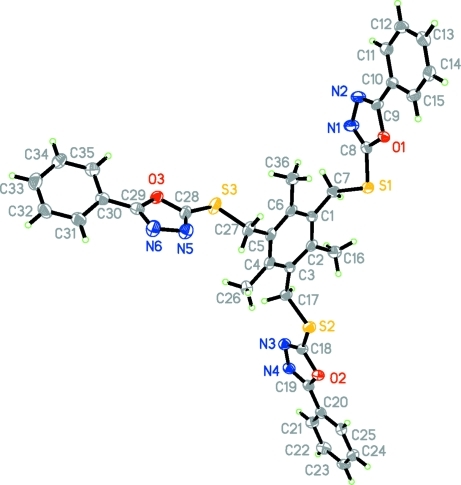
View of (I) showing the atom-labelling scheme. Displacement ellipsoids are drawn at the 50% probability level.

## supplementary crystallographic information

### Comment

1,3,4-Oxadiazole derivatives have wide applications in medicine, industry and coordination chemistry, so they are under intensive studies (Al-Talib *et al.*, 1990; Wang *et al.*, 2005; Zhang *et al.*, 2007). Recently, novel thio-based ligands with multi-armed tripodal geometry were synthesized, and these ligands demonstrated their significance in the supramolecular studies (PrakashaReddy & Pedireddi, 2007 and references therein).  Herewith we present the title compound (I), where the 2,4,6-trimethylbenzene center contains three 5-phenyl-1,3,4-oxadiazol-2-ylsulfanylmethyl arms.

In (I) (Fig.1), two phenyloxadiazole fragments - C10—C15/C8/C9/N1/N2/O1 (A) and C20—C25/C18/C19/N3/N4/O2 (B), respectively - are situated on the one side of the central benzene ring (C1—C6), while the third phenyloxadiazole fragment - C30—C35/C28/C29/N5/N6/O3 (C) - is situated on the other side of the central benzene ring. In A and B, the oxadiazole rings are almost coplanar with the attached phenyl rings forming dihedral angles of 1.5 (2) and 2.4 (2)°, respectively. The terminal phenyl rings are roughly orthogonal to the plane of the central benzene ring with dihedral angles of 82.0 (2), 89.2 (2) and 72.4 (2)°, respectively. The crystal packing exhibits weak intermolecular C—H···N interactions (Table 1).

### Experimental

A suspension of 5-phenyl-1,3,4-oxadiazole-2-thiol (3.0 mmol) and 2,4,6-trimethyl-1,3,5-tribromomethyl benzene (1.0 mmol) in ethanol (10 ml) was stirred at room temperature. The reaction progress was monitored *via* TLC. The resulting precipitate was filtered off, washed with cold ethanol, dried and purified to give the target product as white solid in 80% yield. Crystals of (I) suitable for single-crystal X-ray analysis were grown by slow evaporation of a solution in chloroform-ethanol (1:1).

### Refinement

All H atoms were positioned geometrically and refined as riding (C—H = 0.95–0.99 Å) and allowed to ride on their parent atoms, with *U*_iso_(H) =1.2-1.5*U*_eq_ of the parent atom.

### Crystal data

**Table:**

C_36_H_30_N_6_O_3_S_3_	*F*(000) = 1440
*M**_r_* = 690.84	*D*_x_ = 1.425 Mg m^−^^3^
Monoclinic, *P*2_1_/*c*	Mo *K*α radiation, λ = 0.71073 Å
Hall symbol: -P 2ybc	Cell parameters from 7164 reflections
*a* = 19.870 (4) Å	θ = 2.1–27.9°
*b* = 9.1305 (18) Å	µ = 0.28 mm^−^^1^
*c* = 18.557 (4) Å	*T* = 113 K
β = 107.00 (3)°	Prism, colourless
*V* = 3219.6 (11) Å^3^	0.22 × 0.20 × 0.10 mm
*Z* = 4	

### Data collection

**Table:**

Rigaku Saturn CCD area-detector diffractometer	5664 independent reflections
Radiation source: rotating anode	4490 reflections with *I* > 2σ(*I*)
confocal	*R*_int_ = 0.051
Detector resolution: 7.31 pixels mm^-1^	θ_max_ = 25.0°, θ_min_ = 2.1°
φ and ω scans	*h* = −23→19
Absorption correction: multi-scan (*CrystalClear*; Rigaku/MSC, 1999)	*k* = −10→10
*T*_min_ = 0.941, *T*_max_ = 0.973	*l* = −19→22
23119 measured reflections	

### Refinement

**Table:**

Refinement on *F*^2^	Secondary atom site location: difference Fourier map
Least-squares matrix: full	Hydrogen site location: inferred from neighbouring sites
*R*[*F*^2^ > 2σ(*F*^2^)] = 0.049	H-atom parameters constrained
*wR*(*F*^2^) = 0.149	*w* = 1/[σ^2^(*F*_o_^2^) + (0.0804*P*)^2^ + 0.7757*P*] where *P* = (*F*_o_^2^ + 2*F*_c_^2^)/3
*S* = 1.10	(Δ/σ)_max_ = 0.001
5664 reflections	Δρ_max_ = 0.35 e Å^−^^3^
437 parameters	Δρ_min_ = −0.37 e Å^−^^3^
0 restraints	Extinction correction: *SHELXL97* (Sheldrick, 2008), Fc^*^=kFc[1+0.001xFc^2^λ^3^/sin(2θ)]^-1/4^
Primary atom site location: structure-invariant direct methods	Extinction coefficient: 0.0130 (11)

### Special details

**Table:**

Geometry. All e.s.d.'s (except the e.s.d. in the dihedral angle between two l.s. planes) are estimated using the full covariance matrix. The cell e.s.d.'s are taken into account individually in the estimation of e.s.d.'s in distances, angles and torsion angles; correlations between e.s.d.'s in cell parameters are only used when they are defined by crystal symmetry. An approximate (isotropic) treatment of cell e.s.d.'s is used for estimating e.s.d.'s involving l.s. planes.
Refinement. Refinement of *F*^2^ against ALL reflections. The weighted *R*-factor *wR* and goodness of fit *S* are based on *F*^2^, conventional *R*-factors *R* are based on *F*, with *F* set to zero for negative *F*^2^. The threshold expression of *F*^2^ > σ(*F*^2^) is used only for calculating *R*-factors(gt) *etc*. and is not relevant to the choice of reflections for refinement. *R*-factors based on *F*^2^ are statistically about twice as large as those based on *F*, and *R*- factors based on ALL data will be even larger.

### Fractional atomic coordinates and isotropic or equivalent isotropic displacement parameters (Å^2^)

**Table:**

	*x*	*y*	*z*	*U*_iso_*/*U*_eq_	
S1	0.18609 (4)	0.29928 (8)	0.04709 (4)	0.0293 (2)	
S2	0.17834 (4)	0.61632 (8)	0.36748 (4)	0.0305 (2)	
S3	0.30065 (4)	0.98854 (9)	0.09276 (5)	0.0366 (3)	
O1	0.24745 (9)	0.2057 (2)	−0.05257 (11)	0.0242 (5)	
O2	0.12205 (9)	0.6002 (2)	0.47942 (11)	0.0260 (5)	
O3	0.37150 (9)	1.2260 (2)	0.08047 (11)	0.0274 (5)	
N1	0.16166 (13)	0.3621 (3)	−0.10346 (15)	0.0361 (6)	
N2	0.19355 (13)	0.3179 (3)	−0.15899 (14)	0.0337 (6)	
N3	0.06871 (12)	0.7678 (3)	0.39516 (14)	0.0282 (6)	
N4	0.03554 (12)	0.7624 (3)	0.45304 (14)	0.0278 (6)	
N5	0.43389 (12)	1.0801 (3)	0.17101 (15)	0.0350 (6)	
N6	0.47315 (13)	1.2090 (3)	0.16849 (16)	0.0370 (7)	
C1	0.17176 (14)	0.5716 (3)	0.10460 (16)	0.0237 (6)	
C2	0.14838 (14)	0.5860 (3)	0.16842 (16)	0.0255 (6)	
C3	0.17930 (14)	0.6912 (3)	0.22331 (16)	0.0249 (6)	
C4	0.23874 (15)	0.7703 (3)	0.21951 (17)	0.0276 (7)	
C5	0.26506 (14)	0.7483 (3)	0.15825 (17)	0.0255 (6)	
C6	0.23050 (14)	0.6535 (3)	0.09961 (16)	0.0255 (6)	
C7	0.13485 (14)	0.4691 (3)	0.04168 (17)	0.0279 (7)	
H7A	0.1292	0.5173	−0.0075	0.033*	
H7B	0.0874	0.4455	0.0456	0.033*	
C8	0.19554 (14)	0.2940 (3)	−0.04264 (17)	0.0263 (7)	
C9	0.24297 (14)	0.2274 (3)	−0.12660 (16)	0.0256 (6)	
C10	0.29139 (14)	0.1506 (3)	−0.15903 (16)	0.0250 (6)	
C11	0.28586 (15)	0.1687 (3)	−0.23545 (17)	0.0288 (7)	
H11	0.2513	0.2328	−0.2656	0.035*	
C12	0.33012 (15)	0.0942 (3)	−0.26695 (18)	0.0314 (7)	
H12	0.3259	0.1061	−0.3190	0.038*	
C13	0.38190 (15)	0.0000 (3)	−0.22227 (19)	0.0320 (7)	
H13	0.4130	−0.0509	−0.2439	0.038*	
C14	0.38718 (15)	−0.0176 (3)	−0.14763 (19)	0.0324 (7)	
H14	0.4221	−0.0812	−0.1176	0.039*	
C15	0.34215 (14)	0.0562 (3)	−0.11523 (17)	0.0300 (7)	
H15	0.3460	0.0424	−0.0634	0.036*	
C16	0.08921 (16)	0.4894 (4)	0.17749 (18)	0.0348 (7)	
H16A	0.0438	0.5355	0.1525	0.052*	
H16B	0.0940	0.4768	0.2312	0.052*	
H16C	0.0916	0.3936	0.1546	0.052*	
C17	0.14338 (15)	0.7269 (3)	0.28308 (16)	0.0281 (7)	
H17A	0.0922	0.7089	0.2625	0.034*	
H17B	0.1503	0.8319	0.2965	0.034*	
C18	0.11798 (14)	0.6706 (3)	0.41349 (16)	0.0254 (6)	
C19	0.06834 (13)	0.6633 (3)	0.50062 (16)	0.0245 (6)	
C20	0.05478 (14)	0.6132 (3)	0.56941 (17)	0.0254 (6)	
C21	−0.00131 (14)	0.6715 (3)	0.59100 (17)	0.0298 (7)	
H21	−0.0313	0.7431	0.5606	0.036*	
C22	−0.01283 (15)	0.6236 (4)	0.65760 (18)	0.0346 (8)	
H22	−0.0506	0.6633	0.6730	0.042*	
C23	0.03049 (15)	0.5184 (4)	0.70162 (18)	0.0341 (8)	
H23	0.0223	0.4860	0.7470	0.041*	
C24	0.08569 (16)	0.4605 (3)	0.67948 (17)	0.0327 (7)	
H24	0.1150	0.3875	0.7094	0.039*	
C25	0.09819 (15)	0.5085 (3)	0.61421 (17)	0.0303 (7)	
H25	0.1367	0.4699	0.5998	0.036*	
C26	0.27447 (18)	0.8753 (4)	0.2815 (2)	0.0409 (8)	
H26A	0.3251	0.8553	0.2982	0.061*	
H26B	0.2551	0.8631	0.3240	0.061*	
H26C	0.2664	0.9760	0.2627	0.061*	
C27	0.33018 (15)	0.8295 (3)	0.15422 (19)	0.0318 (7)	
H27A	0.3600	0.7651	0.1334	0.038*	
H27B	0.3580	0.8617	0.2051	0.038*	
C28	0.37511 (15)	1.0980 (3)	0.11886 (17)	0.0287 (7)	
C29	0.43512 (13)	1.2908 (3)	0.11594 (16)	0.0257 (7)	
C30	0.44873 (14)	1.4375 (3)	0.09273 (17)	0.0274 (7)	
C31	0.49555 (15)	1.5280 (4)	0.14417 (18)	0.0338 (7)	
H31	0.5191	1.4929	0.1932	0.041*	
C32	0.50769 (17)	1.6693 (4)	0.1237 (2)	0.0403 (8)	
H32	0.5395	1.7310	0.1589	0.048*	
C33	0.47366 (17)	1.7209 (4)	0.0524 (2)	0.0418 (8)	
H33	0.4816	1.8183	0.0389	0.050*	
C34	0.42781 (16)	1.6304 (4)	0.0003 (2)	0.0369 (8)	
H34	0.4051	1.6652	−0.0491	0.044*	
C35	0.41525 (15)	1.4894 (3)	0.02058 (18)	0.0315 (7)	
H35	0.3837	1.4277	−0.0149	0.038*	
C36	0.25732 (16)	0.6325 (3)	0.03167 (18)	0.0334 (7)	
H36A	0.2810	0.7221	0.0230	0.050*	
H36B	0.2176	0.6114	−0.0128	0.050*	
H36C	0.2906	0.5506	0.0409	0.050*	

### Atomic displacement parameters (Å^2^)

**Table:**

	*U*^11^	*U*^22^	*U*^33^	*U*^12^	*U*^13^	*U*^23^
S1	0.0405 (4)	0.0254 (4)	0.0238 (4)	0.0061 (3)	0.0123 (3)	0.0017 (3)
S2	0.0366 (4)	0.0302 (5)	0.0267 (4)	0.0085 (3)	0.0127 (3)	0.0024 (3)
S3	0.0302 (4)	0.0306 (5)	0.0435 (5)	−0.0040 (3)	0.0020 (3)	0.0140 (4)
O1	0.0283 (10)	0.0238 (11)	0.0189 (11)	0.0006 (8)	0.0043 (8)	−0.0021 (8)
O2	0.0278 (10)	0.0296 (12)	0.0215 (11)	0.0002 (8)	0.0084 (8)	−0.0016 (9)
O3	0.0252 (10)	0.0298 (12)	0.0241 (11)	−0.0036 (8)	0.0022 (8)	0.0055 (9)
N1	0.0448 (15)	0.0405 (16)	0.0248 (15)	0.0129 (12)	0.0130 (12)	0.0037 (12)
N2	0.0395 (14)	0.0385 (16)	0.0229 (15)	0.0066 (12)	0.0090 (11)	0.0008 (12)
N3	0.0298 (13)	0.0302 (14)	0.0271 (14)	0.0007 (11)	0.0119 (11)	0.0008 (11)
N4	0.0277 (12)	0.0319 (14)	0.0260 (14)	−0.0018 (10)	0.0115 (11)	−0.0036 (11)
N5	0.0285 (13)	0.0358 (15)	0.0357 (16)	−0.0010 (11)	0.0016 (11)	0.0076 (12)
N6	0.0297 (13)	0.0354 (16)	0.0393 (17)	−0.0042 (11)	−0.0002 (12)	0.0057 (13)
C1	0.0279 (14)	0.0201 (15)	0.0227 (16)	0.0079 (11)	0.0067 (12)	0.0022 (12)
C2	0.0277 (14)	0.0237 (15)	0.0257 (16)	0.0065 (12)	0.0086 (12)	0.0074 (13)
C3	0.0317 (15)	0.0202 (15)	0.0232 (16)	0.0068 (12)	0.0088 (12)	0.0009 (12)
C4	0.0338 (16)	0.0207 (15)	0.0291 (17)	0.0035 (12)	0.0105 (13)	0.0036 (12)
C5	0.0276 (15)	0.0192 (15)	0.0304 (17)	0.0051 (11)	0.0092 (12)	0.0086 (12)
C6	0.0324 (15)	0.0224 (15)	0.0230 (16)	0.0095 (12)	0.0099 (12)	0.0084 (12)
C7	0.0286 (15)	0.0305 (17)	0.0233 (16)	0.0060 (12)	0.0057 (12)	0.0004 (13)
C8	0.0304 (15)	0.0214 (15)	0.0270 (17)	0.0016 (12)	0.0081 (12)	−0.0017 (12)
C9	0.0300 (15)	0.0259 (16)	0.0184 (16)	−0.0019 (12)	0.0035 (12)	0.0032 (12)
C10	0.0285 (15)	0.0249 (16)	0.0241 (16)	−0.0069 (12)	0.0114 (12)	−0.0028 (12)
C11	0.0299 (15)	0.0286 (16)	0.0252 (17)	−0.0032 (12)	0.0037 (12)	−0.0016 (13)
C12	0.0375 (16)	0.0338 (18)	0.0261 (17)	−0.0087 (13)	0.0143 (13)	−0.0032 (13)
C13	0.0282 (15)	0.0321 (18)	0.042 (2)	−0.0063 (13)	0.0195 (14)	−0.0083 (14)
C14	0.0343 (16)	0.0253 (17)	0.0368 (19)	−0.0026 (12)	0.0093 (14)	0.0030 (14)
C15	0.0319 (15)	0.0325 (17)	0.0249 (17)	−0.0028 (13)	0.0074 (12)	0.0021 (13)
C16	0.0392 (17)	0.0366 (19)	0.0297 (18)	−0.0024 (14)	0.0119 (14)	−0.0007 (14)
C17	0.0323 (15)	0.0288 (16)	0.0230 (16)	0.0051 (12)	0.0078 (12)	0.0040 (13)
C18	0.0275 (14)	0.0264 (16)	0.0219 (16)	−0.0023 (12)	0.0066 (12)	−0.0007 (12)
C19	0.0217 (14)	0.0261 (16)	0.0252 (16)	−0.0056 (11)	0.0063 (12)	−0.0053 (12)
C20	0.0246 (14)	0.0247 (16)	0.0278 (17)	−0.0064 (11)	0.0092 (12)	−0.0040 (12)
C21	0.0225 (14)	0.0382 (18)	0.0264 (17)	−0.0013 (12)	0.0037 (12)	0.0014 (14)
C22	0.0252 (15)	0.048 (2)	0.0335 (19)	−0.0034 (14)	0.0135 (13)	−0.0036 (15)
C23	0.0367 (17)	0.042 (2)	0.0220 (17)	−0.0119 (14)	0.0065 (13)	−0.0025 (14)
C24	0.0372 (17)	0.0315 (17)	0.0264 (18)	−0.0010 (13)	0.0045 (13)	0.0038 (14)
C25	0.0358 (16)	0.0246 (16)	0.0304 (18)	−0.0012 (12)	0.0095 (13)	−0.0024 (13)
C26	0.051 (2)	0.0265 (18)	0.044 (2)	−0.0055 (14)	0.0128 (16)	−0.0038 (15)
C27	0.0309 (15)	0.0277 (17)	0.0378 (19)	0.0045 (13)	0.0114 (13)	0.0111 (14)
C28	0.0322 (16)	0.0238 (16)	0.0308 (17)	0.0017 (12)	0.0104 (13)	0.0044 (13)
C29	0.0188 (13)	0.0333 (17)	0.0233 (16)	0.0011 (11)	0.0037 (11)	−0.0025 (13)
C30	0.0241 (14)	0.0329 (17)	0.0266 (17)	−0.0024 (12)	0.0096 (12)	−0.0024 (13)
C31	0.0313 (16)	0.0394 (19)	0.0308 (18)	−0.0061 (13)	0.0090 (13)	−0.0066 (14)
C32	0.0437 (18)	0.039 (2)	0.040 (2)	−0.0163 (15)	0.0159 (16)	−0.0134 (16)
C33	0.0451 (19)	0.0347 (19)	0.051 (2)	−0.0057 (15)	0.0221 (17)	0.0033 (16)
C34	0.0353 (17)	0.040 (2)	0.038 (2)	−0.0031 (14)	0.0156 (14)	0.0073 (15)
C35	0.0251 (15)	0.0378 (18)	0.0311 (18)	−0.0026 (13)	0.0077 (13)	−0.0009 (14)
C36	0.0419 (17)	0.0302 (17)	0.0327 (19)	0.0070 (13)	0.0182 (14)	0.0055 (14)

### Geometric parameters (Å, °)

**Table:**

S1—C8	1.730 (3)	C13—C14	1.368 (4)
S1—C7	1.842 (3)	C13—H13	0.9500
S2—C18	1.736 (3)	C14—C15	1.390 (4)
S2—C17	1.822 (3)	C14—H14	0.9500
S3—C28	1.733 (3)	C15—H15	0.9500
S3—C27	1.833 (3)	C16—H16A	0.9800
O1—C8	1.364 (3)	C16—H16B	0.9800
O1—C9	1.365 (3)	C16—H16C	0.9800
O2—C18	1.364 (3)	C17—H17A	0.9900
O2—C19	1.368 (3)	C17—H17B	0.9900
O3—C28	1.360 (3)	C19—C20	1.453 (4)
O3—C29	1.376 (3)	C20—C25	1.389 (4)
N1—C8	1.292 (4)	C20—C21	1.395 (4)
N1—N2	1.417 (4)	C21—C22	1.392 (4)
N2—C9	1.290 (4)	C21—H21	0.9500
N3—C18	1.291 (4)	C22—C23	1.386 (4)
N3—N4	1.416 (3)	C22—H22	0.9500
N4—C19	1.298 (4)	C23—C24	1.384 (4)
N5—C28	1.291 (4)	C23—H23	0.9500
N5—N6	1.421 (4)	C24—C25	1.377 (4)
N6—C29	1.284 (4)	C24—H24	0.9500
C1—C2	1.399 (4)	C25—H25	0.9500
C1—C6	1.412 (4)	C26—H26A	0.9800
C1—C7	1.509 (4)	C26—H26B	0.9800
C2—C3	1.404 (4)	C26—H26C	0.9800
C2—C16	1.518 (4)	C27—H27A	0.9900
C3—C4	1.403 (4)	C27—H27B	0.9900
C3—C17	1.520 (4)	C29—C30	1.457 (4)
C4—C5	1.398 (4)	C30—C35	1.392 (4)
C4—C26	1.507 (4)	C30—C31	1.393 (4)
C5—C6	1.403 (4)	C31—C32	1.385 (5)
C5—C27	1.512 (4)	C31—H31	0.9500
C6—C36	1.518 (4)	C32—C33	1.382 (5)
C7—H7A	0.9900	C32—H32	0.9500
C7—H7B	0.9900	C33—C34	1.390 (5)
C9—C10	1.455 (4)	C33—H33	0.9500
C10—C15	1.393 (4)	C34—C35	1.384 (4)
C10—C11	1.400 (4)	C34—H34	0.9500
C11—C12	1.371 (4)	C35—H35	0.9500
C11—H11	0.9500	C36—H36A	0.9800
C12—C13	1.409 (4)	C36—H36B	0.9800
C12—H12	0.9500	C36—H36C	0.9800
			
C8—S1—C7	100.65 (14)	C3—C17—H17B	109.3
C18—S2—C17	96.89 (14)	S2—C17—H17B	109.3
C28—S3—C27	101.01 (14)	H17A—C17—H17B	108.0
C8—O1—C9	102.7 (2)	N3—C18—O2	113.7 (2)
C18—O2—C19	102.3 (2)	N3—C18—S2	130.3 (2)
C28—O3—C29	102.6 (2)	O2—C18—S2	116.0 (2)
C8—N1—N2	105.6 (2)	N4—C19—O2	112.1 (2)
C9—N2—N1	106.5 (2)	N4—C19—C20	129.1 (3)
C18—N3—N4	105.1 (2)	O2—C19—C20	118.7 (2)
C19—N4—N3	106.7 (2)	C25—C20—C21	119.9 (3)
C28—N5—N6	104.8 (2)	C25—C20—C19	120.0 (3)
C29—N6—N5	107.5 (2)	C21—C20—C19	120.1 (3)
C2—C1—C6	119.4 (3)	C22—C21—C20	119.2 (3)
C2—C1—C7	120.4 (3)	C22—C21—H21	120.4
C6—C1—C7	120.1 (3)	C20—C21—H21	120.4
C1—C2—C3	119.7 (3)	C23—C22—C21	120.4 (3)
C1—C2—C16	119.9 (3)	C23—C22—H22	119.8
C3—C2—C16	120.4 (3)	C21—C22—H22	119.8
C4—C3—C2	120.8 (3)	C24—C23—C22	120.0 (3)
C4—C3—C17	120.5 (3)	C24—C23—H23	120.0
C2—C3—C17	118.5 (3)	C22—C23—H23	120.0
C5—C4—C3	119.2 (3)	C25—C24—C23	120.1 (3)
C5—C4—C26	120.5 (3)	C25—C24—H24	119.9
C3—C4—C26	120.3 (3)	C23—C24—H24	119.9
C4—C5—C6	120.3 (3)	C24—C25—C20	120.3 (3)
C4—C5—C27	119.9 (3)	C24—C25—H25	119.8
C6—C5—C27	119.8 (3)	C20—C25—H25	119.8
C5—C6—C1	120.2 (3)	C4—C26—H26A	109.5
C5—C6—C36	120.7 (3)	C4—C26—H26B	109.5
C1—C6—C36	119.1 (3)	H26A—C26—H26B	109.5
C1—C7—S1	110.14 (19)	C4—C26—H26C	109.5
C1—C7—H7A	109.6	H26A—C26—H26C	109.5
S1—C7—H7A	109.6	H26B—C26—H26C	109.5
C1—C7—H7B	109.6	C5—C27—S3	107.24 (19)
S1—C7—H7B	109.6	C5—C27—H27A	110.3
H7A—C7—H7B	108.1	S3—C27—H27A	110.3
N1—C8—O1	112.8 (3)	C5—C27—H27B	110.3
N1—C8—S1	130.7 (2)	S3—C27—H27B	110.3
O1—C8—S1	116.5 (2)	H27A—C27—H27B	108.5
N2—C9—O1	112.3 (3)	N5—C28—O3	113.5 (2)
N2—C9—C10	128.4 (3)	N5—C28—S3	130.4 (2)
O1—C9—C10	119.3 (2)	O3—C28—S3	116.1 (2)
C15—C10—C11	119.5 (3)	N6—C29—O3	111.6 (3)
C15—C10—C9	120.7 (3)	N6—C29—C30	129.8 (3)
C11—C10—C9	119.8 (3)	O3—C29—C30	118.5 (2)
C12—C11—C10	120.2 (3)	C35—C30—C31	119.5 (3)
C12—C11—H11	119.9	C35—C30—C29	121.3 (3)
C10—C11—H11	119.9	C31—C30—C29	119.2 (3)
C11—C12—C13	120.1 (3)	C32—C31—C30	120.0 (3)
C11—C12—H12	120.0	C32—C31—H31	120.0
C13—C12—H12	120.0	C30—C31—H31	120.0
C14—C13—C12	119.6 (3)	C33—C32—C31	120.3 (3)
C14—C13—H13	120.2	C33—C32—H32	119.9
C12—C13—H13	120.2	C31—C32—H32	119.9
C13—C14—C15	120.8 (3)	C32—C33—C34	120.1 (3)
C13—C14—H14	119.6	C32—C33—H33	120.0
C15—C14—H14	119.6	C34—C33—H33	120.0
C14—C15—C10	119.7 (3)	C35—C34—C33	119.8 (3)
C14—C15—H15	120.1	C35—C34—H34	120.1
C10—C15—H15	120.1	C33—C34—H34	120.1
C2—C16—H16A	109.5	C34—C35—C30	120.3 (3)
C2—C16—H16B	109.5	C34—C35—H35	119.9
H16A—C16—H16B	109.5	C30—C35—H35	119.9
C2—C16—H16C	109.5	C6—C36—H36A	109.5
H16A—C16—H16C	109.5	C6—C36—H36B	109.5
H16B—C16—H16C	109.5	H36A—C36—H36B	109.5
C3—C17—S2	111.49 (19)	C6—C36—H36C	109.5
C3—C17—H17A	109.3	H36A—C36—H36C	109.5
S2—C17—H17A	109.3	H36B—C36—H36C	109.5
			
C8—N1—N2—C9	0.4 (3)	C9—C10—C15—C14	179.0 (3)
C18—N3—N4—C19	0.4 (3)	C4—C3—C17—S2	−91.9 (3)
C28—N5—N6—C29	−0.1 (3)	C2—C3—C17—S2	93.2 (3)
C6—C1—C2—C3	5.9 (4)	C18—S2—C17—C3	−172.6 (2)
C7—C1—C2—C3	−174.4 (2)	N4—N3—C18—O2	−0.8 (3)
C6—C1—C2—C16	−174.9 (2)	N4—N3—C18—S2	178.8 (2)
C7—C1—C2—C16	4.8 (4)	C19—O2—C18—N3	0.8 (3)
C1—C2—C3—C4	−7.5 (4)	C19—O2—C18—S2	−178.82 (18)
C16—C2—C3—C4	173.3 (3)	C17—S2—C18—N3	−5.2 (3)
C1—C2—C3—C17	167.3 (2)	C17—S2—C18—O2	174.3 (2)
C16—C2—C3—C17	−11.9 (4)	N3—N4—C19—O2	0.1 (3)
C2—C3—C4—C5	3.0 (4)	N3—N4—C19—C20	−179.1 (3)
C17—C3—C4—C5	−171.7 (3)	C18—O2—C19—N4	−0.5 (3)
C2—C3—C4—C26	−175.8 (3)	C18—O2—C19—C20	178.8 (2)
C17—C3—C4—C26	9.5 (4)	N4—C19—C20—C25	−177.6 (3)
C3—C4—C5—C6	3.0 (4)	O2—C19—C20—C25	3.2 (4)
C26—C4—C5—C6	−178.2 (3)	N4—C19—C20—C21	1.6 (5)
C3—C4—C5—C27	−178.4 (2)	O2—C19—C20—C21	−177.6 (2)
C26—C4—C5—C27	0.4 (4)	C25—C20—C21—C22	0.1 (4)
C4—C5—C6—C1	−4.5 (4)	C19—C20—C21—C22	−179.1 (3)
C27—C5—C6—C1	176.8 (2)	C20—C21—C22—C23	−0.5 (4)
C4—C5—C6—C36	178.0 (3)	C21—C22—C23—C24	0.1 (5)
C27—C5—C6—C36	−0.6 (4)	C22—C23—C24—C25	0.8 (5)
C2—C1—C6—C5	0.1 (4)	C23—C24—C25—C20	−1.2 (5)
C7—C1—C6—C5	−179.7 (2)	C21—C20—C25—C24	0.8 (4)
C2—C1—C6—C36	177.5 (2)	C19—C20—C25—C24	−180.0 (3)
C7—C1—C6—C36	−2.2 (4)	C4—C5—C27—S3	−98.1 (3)
C2—C1—C7—S1	−102.4 (3)	C6—C5—C27—S3	80.5 (3)
C6—C1—C7—S1	77.3 (3)	C28—S3—C27—C5	160.8 (2)
C8—S1—C7—C1	−125.3 (2)	N6—N5—C28—O3	1.1 (3)
N2—N1—C8—O1	−0.7 (3)	N6—N5—C28—S3	−176.8 (3)
N2—N1—C8—S1	178.4 (2)	C29—O3—C28—N5	−1.5 (3)
C9—O1—C8—N1	0.8 (3)	C29—O3—C28—S3	176.7 (2)
C9—O1—C8—S1	−178.54 (19)	C27—S3—C28—N5	−4.7 (3)
C7—S1—C8—N1	−17.2 (3)	C27—S3—C28—O3	177.5 (2)
C7—S1—C8—O1	161.9 (2)	N5—N6—C29—O3	−0.8 (3)
N1—N2—C9—O1	0.1 (3)	N5—N6—C29—C30	176.3 (3)
N1—N2—C9—C10	180.0 (3)	C28—O3—C29—N6	1.4 (3)
C8—O1—C9—N2	−0.5 (3)	C28—O3—C29—C30	−176.1 (3)
C8—O1—C9—C10	179.6 (2)	N6—C29—C30—C35	157.2 (3)
N2—C9—C10—C15	179.8 (3)	O3—C29—C30—C35	−25.9 (4)
O1—C9—C10—C15	−0.3 (4)	N6—C29—C30—C31	−23.5 (5)
N2—C9—C10—C11	−1.8 (5)	O3—C29—C30—C31	153.4 (3)
O1—C9—C10—C11	178.1 (2)	C35—C30—C31—C32	1.0 (5)
C15—C10—C11—C12	−0.1 (4)	C29—C30—C31—C32	−178.3 (3)
C9—C10—C11—C12	−178.4 (3)	C30—C31—C32—C33	−0.2 (5)
C10—C11—C12—C13	−0.6 (4)	C31—C32—C33—C34	−1.0 (5)
C11—C12—C13—C14	0.7 (4)	C32—C33—C34—C35	1.3 (5)
C12—C13—C14—C15	−0.1 (4)	C33—C34—C35—C30	−0.4 (5)
C13—C14—C15—C10	−0.6 (4)	C31—C30—C35—C34	−0.7 (4)
C11—C10—C15—C14	0.7 (4)	C29—C30—C35—C34	178.6 (3)

### Hydrogen-bond geometry (Å, °)

**Table:**

*D*—H···*A*	*D*—H	H···*A*	*D*···*A*	*D*—H···*A*
C31—H31···N5^i^	0.95	2.55	3.338 (3)	141
C24—H24···N2^ii^	0.95	2.57	3.394 (4)	146

Symmetry codes: (i) −*x*+1, *y*+1/2, −*z*+1/2; (ii) *x*, *y*, *z*+1.
